# ﻿Phylogenetic and evolutionary insights from 30 newly-assembled *Onygenales* Mitochondrial Genomes: co-evolution of introns and HEGs shapes mitogenome size variation

**DOI:** 10.3897/imafungus.16.150451

**Published:** 2025-07-17

**Authors:** Héctor Antônio Assunção Romão, Thalison Rodrigues Moreira, Leonardo Carlos Jeronimo Corvalán, Amanda Alves de Melo-Ximenes, Alexandre Melo Bailão, Clayton Luiz Borges, Renata de Oliveira Dias, Rhewter Nunes

**Affiliations:** 1 Laboratório de Genética & Biodiversidade, Instituto de Ciências Biológicas I, Universidade Federal de Goiás, Goiânia – Goiás, Brazil; 2 Laboratório de Biologia Molecular, Instituto de Ciências Biológicas II, Universidade Federal de Goiás, Goiânia – Goiás, Brazil; 3 Laboratório de Bioinformática e Biodiversidade, Instituto Acadêmico de Ciências da Saúde e Biológicas, Universidade Estadual de Goiás - Campus Oeste - UnU de Iporá, Iporá, Goiás, Brazil

**Keywords:** Comparative genomics, dermatophyte fungi, evolution, phylogenetic signal, structural variation

## Abstract

Mitochondrial genomes (mtDNA) provide valuable resources for investigating fungal evolution; however, comprehensive mitogenomic datasets for *Onygenales* are still scarce. Here, we assembled and annotated 30 new mitogenomes representing 18 species across five families, substantially expanding the available resources for this order. We tested two evolutionary hypotheses: (1) that structural features of mitochondrial genomes are phylogenetically conserved and (2) that introns and homing endonuclease genes (HEGs) have co-evolved and contributed to genome size variation. All mitogenomes exhibited conserved protein-coding content, but showed considerable variation in intron number and genome size. Phylogenetic signal was significant for multiple traits, including gene number and intron abundance. Furthermore, phylogenetic regression analyses revealed a strong correlation between intron content and HEG abundance, thereby substantiating the hypothesis of coordinated evolution. Our findings demonstrate that mitochondrial genome evolution in *Onygenales* reflects both structural conservation and lineage-specific expansion patterns, shaped in part by the distribution of introns and HEGs.

## ﻿Introduction

The order *Onygenales* encompasses a phylogenetically diverse assemblage of species occupying a broad range of ecological niches, including saprophytic, entomopathogenic and mammalian-associated environments. These taxa exhibit a wide range of adaptive strategies, including osmophily, thermophily, cellulolytic and keratinolytic capabilities, as well as trophic preferences spanning eutrophic and oligotrophic conditions, alongside thermal dimorphism (Kandemir et al. 2022). This remarkable diversity of morphological, ecological and physiological traits exhibited by this group presents significant challenges to taxonomic identification, complicates phylogenetic reconstruction and highlights the need to elucidate the genetic mechanisms underlying their adaptation and diversification.

Mitochondria, ubiquitous organelles found in nearly all eukaryotic cells, play a pivotal role in cellular energy production and various metabolic processes (Herst et al. 2017). These organelles are crucial for ATP production through oxidative phosphorylation and are essential in the biosynthesis of macromolecules, including nucleotides, lipids, heme and iron-sulphur clusters (Vakifahmetoglu-Norberg et al. 2017). Fungal mitochondrial genomes typically encode 15 proteins that are essential for cellular respiration, ribosomal RNA genes, 20 to 30 tRNAs, non-coding regions and mobile elements, such as introns and homing endonuclease genes (HEGs) (Aguileta et al. 2014; Sandor et al. 2018; Fonseca et al. 2020). The variation in the presence and number of these elements can significantly impact genome architecture and evolutionary patterns. The use of mitochondrial genomic data for phylogenetic studies in fungi is not novel, as it has been successfully employed to investigate evolutionary relationships within the order *Onygenales* (Teixeira et al. 2021). While mitogenomes effectively resolved species-level relationships in this group, they proved insufficient for delineating intraspecific dynamics within the *Coccidioides* genus. Similarly, in *Eurotiomycetes*, mitochondrial protein-coding genes yielded well-supported topologies for *Eurotiales* in both Bayesian and Maximum Likelihood analyses (Zhang et al. 2024). Collectively, these studies underscore the utility of mitogenomes as a valuable resource for resolving intraordinal phylogenetic relationships. Furthermore, recent studies, such as Megarioti and Kouvelis (2020), have proposed that introns and HEGs may co-evolve and that this functional association contributes to mitochondrial genome expansion in fungi. However, this hypothesis was based on only four *Onygenales* mitogenomes, limiting its generalisability within the order. This study addresses this gap by assembling, curating and comparatively analysing 30 mitochondrial genomes from this fungal order. The objective is to identify the factors that shape mitochondrial genome evolution and to test hypotheses related to structural conservation and intron–HEG co-evolution.

This extensive array of unique features inherent to fungal mitochondrial genomes provides a valuable framework for comparative genomic analyses. The objective of this study is twofold: (i) to expand and curate the available mitochondrial genomic resources for the *Onygenales* order by leveraging underexplored public datasets and (ii) to investigate which genomic features have shaped the evolutionary trajectories of mitochondrial genomes within *Onygenales*.

To guide this investigation, we formulated two complementary evolutionary hypotheses. The first hypothesis focuses on structural conservation and proposes that certain genomic traits (e.g. the number of protein-coding genes and the number of tRNAs) are phylogenetically conserved across *Onygenales* lineages. This leads to the following questions: Which structural traits exhibit the strongest phylogenetic signal (as measured by Blomberg’s K and Pagel’s λ)? Are these traits conserved across major clades within *Onygenales*? Is mitochondrial genome size associated with the stability of these structural features?

The second hypothesis addresses functional expansion and postulates that the accumulation of introns and homing endonuclease genes (HEGs) underlies mitochondrial genome expansion in *Onygenales*. It is hypothesised that these elements have co-evolved in a phylogenetically structured, though evolutionarily labile, pattern. In light of these observations, the central question guiding this study is as follows: Is there a significant correlation between the number of introns and HEGs when phylogenetic relationships are accounted for? Does intron content, HEG abundance or intron-rich regions explain the observed variation in mitochondrial genome size? Additionally, are these traits randomly distributed or do they follow phylogenetically structured patterns across lineages?

## ﻿Materials and methods

### ﻿Mitochondrial genome assembly and annotation

The raw Illumina paired-end reads utilised in this study were obtained from the National Center for Biotechnological Information (NCBI) Sequence Read Archive (SRA) database (https://www.ncbi.nlm.nih.gov/sra), in a search performed in March of 2023, retrieving only sequences from species with no complete mitochondrial record in GenBank database (Suppl. material 1). The collected raw data underwent a selection process that prioritised the largest dataset from each strain, based on the total base sizes of the experiments. The mitochondrial genome assembly was performed using NOVOPlasty 4.3.1 software (Dierckxsens et al. 2017), employing both forward and reverse reads. For the beginning of the assembly process, the complete protein-coding sequences (CDS) of the cytochrome oxidase subunit (*COX1*) gene for the species *Trichophytonrubrum* (syn. *T. kuryangei IHEM 26527*) (MW464866.1), *Epidermophytonfloccosum* (NC_007394.1), *Microsporumcanis* (NC_012832.1), *Coccidioidesposadasii* strain Tuc2 (NC_058290.1) and *Coccidioidesimmitis* strain WA221 (NC_058291.1) were used as seed. The sequences utilised for each assembly are described in Suppl. material 2. All the parameters were standardised to k-mer values of 33 and both read and insert sizes were adjusted, based on the dataset in the SRA Experiments database for each species. Following the genome assembly, AWA (Machado et al. 2018) was employed to evaluate the circularisation integrality of the obtained complete genomes. Genomes were classified as correctly circularised if the match score exceeded 95% and had a minimum alignment score of -2. The AWA results for all assemblies are described in Suppl. material 3.

The fungal complete mitogenome was annotated using the MITOS2 web server (Bernt et al. 2013; Donath et al. 2019), according to RefSeq 89 standards. Additionally, the MFannot pipeline (https://megasun.bch.umontreal.ca/apps/mfannot/) was employed to improve the intron boundaries annotation. For both methods, the annotators followed the genetic code for Mold, Protozoan and Coelenterate Mitochondrial (NCBI transl_table=4) for codon usage. Hypothetical open reading frames (ORFs) were identified using the ORF finder tool (https://www.ncbi.nlm.nih.gov/orffinder/). All tRNA gene annotations were refined using tRNAscan-SE 2.0 (Chan and Lowe 2019). In addition, we manually established boundaries of ribosomal genes and determined start and stop codon positions for protein-coding genes (PCGs) using Ugene (Okonechnikov et al. 2012), guided by a sequence alignment against reference genomes retrieved from the RefSeq database. The largest and smallest mitogenomes were selected from the assembled mitochondrial genomes to create graphical representations, plotted using the Chloroplot package version 0.2.4 (Zheng et al. 2020).

### ﻿Genomic comparison

A comparative analysis was conducted on the 30 new mitochondrial genomes and 11 *Onygenales* mitogenomes obtained from the NCBI RefSeq database. The sizes of the coding and non-coding regions and the entire mitogenome were quantitatively compared, as were the number of genes, introns and homing endonuclease-like elements. Nucleotide characterisation was also performed by calculating the AT and GC skew according to the formula: AT-skew = (A - T) / (A + T) and GC-skew = (G - C) / (G + C) for the genome and genetic elements.

To identify the mutation hotspots, the complete sequences of the 15 protein-coding genes (CDS) were extracted from the 41 *Onygenales* genomes analysed and then individually aligned using the multiple sequence aligner MAFFT v.7 (Katoh and Standley 2013). The aligned CDSs were concatenated using SequenceMatrix (Vaidya et al. 2011). The degree of nucleotide diversity between these sequences was estimated using DnaSP v.6.12.0, of which reports the most diverse regions by analysing the positions in the alignment with greatest polymorphism of nucleotide composition (Rozas et al. 2017) and then graphically visualised using the *ggplot2* package in R.

### ﻿Phylogenetic analysis

The 41 *Onygenales* mitogenomes and four outgroup species from the order *Eurotiales*, namely *Aspergillusfumigatus* (NC_017016.1), *Aspergillusluchuensis* (NC_040166.1), *Paecilomycesvariotii* (NC_068093.1) and *Talaromycesmarneffei* (NC_005256.1), were used in the phylogenetic analysis. The amino-acid sequences of the PCGs were collected and individually aligned using MAFFT v.7 under the L-INS-i strategy (Katoh and Standley 2013). The amino-acid alignment was then used as a guide for codon alignment using the Pal2Nal v. 14 software (Suyama et al. 2006). Subsequently, all non-informative positions were removed from the codon alignments using trimAL v.1.4 with the -automated1 option (Capella-Gutiérrez et al. 2009). The resulting PCG codon alignments were finally concatenated using the catfasta2phyml script (https://github.com/nylander/catfasta2phyml).

Phylogenetic reconstruction was performed by Maximum Likelihood using the IQtree v. 1.6.12 software with 1,000 bootstrap replicates (Nguyen et al. 2015). To determine the most suitable nucleotide substitution model, we used the built-in ModelFinder parameter (Kalyaanamoorthy et al. 2017). The biological information related to host, morphology and infection was researched from scientific publications as listed in Suppl. material 4. The tree topology, genomic composition and this biological feature of the species were visualised using the ggtree package available in R (Yu et al. 2017).

### ﻿Comparative phylogenetic analyses

The phylogenetic signal in mitochondrial genome traits was evaluated using Blomberg’s K (Blomberg et al. 2003) and Pagel’s λ (Pagel 1999), applying both statistics to a mitochondrial phylogeny of *Onygenales*. Analyses were conducted in R using the packages phytools (Revell 2012), picante (Kembel et al. 2010) and geiger (Pennell et al. 2014). The traits analysed included total mitochondrial genome size (genome_size), number of protein-coding genes (n_pcg), number of tRNAs (n_trna), number of rRNAs (n_rrna), number of total genes (n_genes), number of introns (n_introns), number of homing endonuclease genes (n_hegs) and the following genomic proportions: coding portion, non-coding portion, portion of protein-coding regions (portion_pcg), portion of tRNAs (portion_trna), portion of rRNAs (portion_rrna) and portion of intronic regions (portion_intron). These values were derived using a custom Python script that parses annotated GenBank files using Biopython libraries (Bio.SeqIO, Bio.SeqUtils, pandas). The script systematically iterates over GenBank entries, categorising annotated features by type (e.g. CDS, tRNA, rRNA, intron) and sums their lengths. The non-coding portion was calculated as the remainder of the genome not annotated as any of the above features.

To assess the evolutionary relationships amongst the aforementioned traits, we performed Phylogenetic Generalised Least Squares (PGLS) regressions using the caper package (Orme et al. 2013), incorporating the phylogenetic co-variance structure derived from the mitogenome tree. PGLS regressions were used to test associations between structural traits of the mitochondrial genomes while accounting for phylogenetic relatedness. In each model, one trait was specified as the response variable and another as the predictor. For example, genome size was modelled as a function of the number of introns and the number of introns was modelled as a function of the number of homing endonuclease genes (HEGs). All PGLS models were fitted using the caper package in R and Pagel’s λ was estimated via Maximum Likelihood for each regression. This approach allows the residual error structure to reflect the degree of phylogenetic signal in the traits being modelled. R² values and p-values for each model are reported in the respective figure panels to indicate model fit and statistical significance. In addition, we applied Mantel tests to evaluate the correlation between pairwise phylogenetic distances (co-phenetic matrix) and trait-based Euclidean distances, using the vegan package (Oksanen et al. 2022). Prior to analysis, all trait values were standardised and traits with zero variance were excluded. Standardisation of trait values prior to multivariate analyses was conducted to homogenise scales and variances across different genomic metrics. This prevents dominance of high-magnitude traits and ensures balanced contributions to distance calculations and ordination axes. For Mantel tests, Euclidean distance matrices were constructed, based on standardised (z-score transformed) trait values across species. The distance matrix reflects a multivariate comparison of all traits considered and was compared against patristic distances extracted from the mitochondrial tree using the ape package in R. Detailed results of phylogenetic signal, PGLS regressions and Mantel tests are provided in Suppl. material 9.

### ﻿Gene selection and mitochondrial gene re-arrangements

The selective pressure for each PCG was calculated using the ratio of non-synonymous mutations (Dn) to synonymous mutations (Ds), such that ω = Dn/Ds. This ratio indicates positive selection when ω > 1, negative selection when ω < 1 and neutrality when ω = 1. The same dataset used in the phylogenetic analysis was used for this analysis, except by the outgroup. The ω was calculated using the null model considering the phylogenetic tree (runmode = 0; model = 0; NSsites = 0) and for the paired relationships using only the null model (runmode = − 2; model = 0; NSsites = 0), both using the PAML 4.9 software (Yang 2007).

Due to the variation in gene numbers, only protein-coding genes were used to analyse gene re-arrangements amongst the 41 *Onygenales* mitochondrial genomes using TreeRex v.1.85 (Bernt et al. 2008). The software was then employed to identify the ancestral gene order and the transposition, inverse transposition, inversion and tandem duplication random loss (TDRL) using the 15 protein-coding genes conserved in all the genomes.

## ﻿Results

### ﻿The characteristics of the 30 newly-assembled *Onygenales* mitogenomes

**Figure 1.**
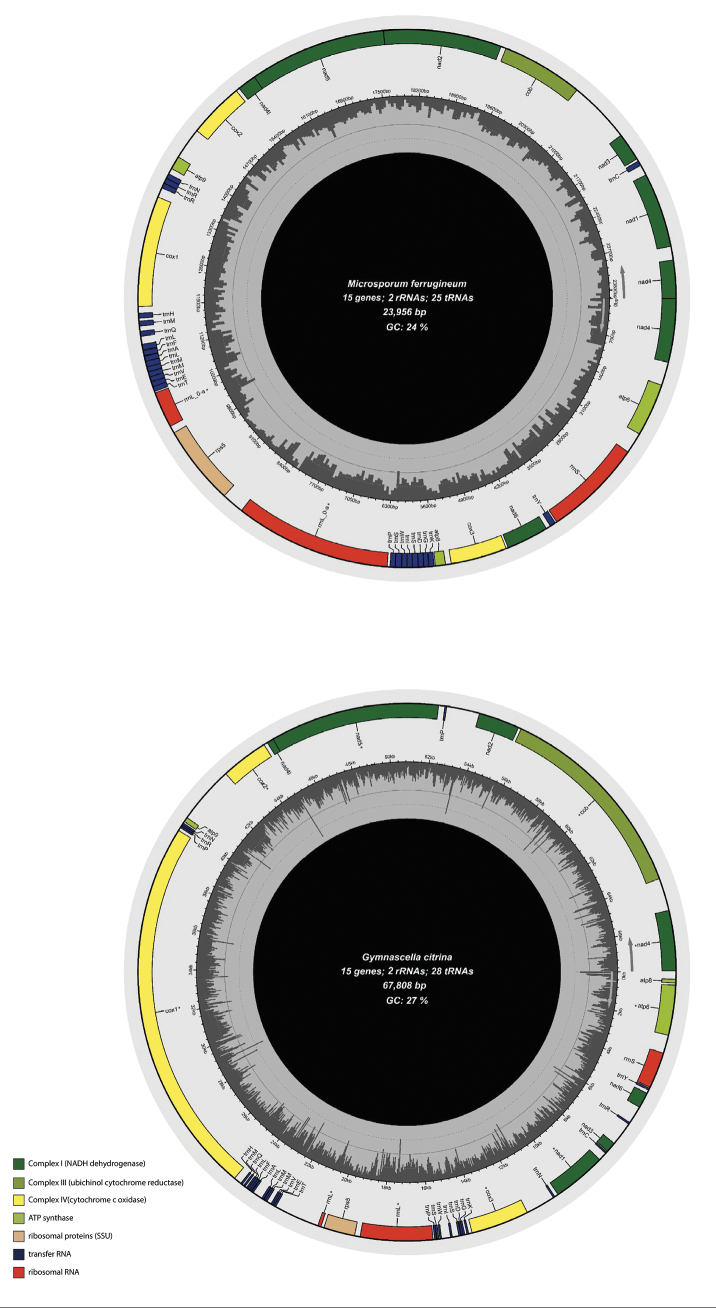
Comparative schematic maps of the assembled mitochondrial genomes of *Microsporumferrugineum* (shortest assembled mitogenome) and *Gymnascellacitrina* (largest assembled mitogenome). The central graphical panel displays GC content distribution across both mitogenomes, with a concentric circle indicating the 50% compositional baseline. Annotated structural features include asterisks (*) denoting intron-containing genes.

The complete circular mitochondrial genome of 30 unprecedented representatives of *Onygenales* was obtained. These mitogenomes included two species that other groups had assembled, but we did not find a reference annotation in the NCBI database. The assemblies ranged from 23,956 (*Microsporumcanis* strain ATCC 36299) to 67,808 bp (*Gymnascellacitrina* strain NRRL 5970) and their GC content varied from 23 to 27% (Fig. 1). Quantitative aspects of the 41 *Onygenales* mitogenomes analysed in this work are described in Suppl. material 5. The data collected includes 18 species: *Blastomycesdermatitidis* (3 strains), *Blastomycesgilchristii*, *Blastomycesparvus*, *Blastomycespercursus*, *Emmonsiacrescens*, *Gymnascellaaurantiaca* NRRL 5967, *Gymnascellacitrina* NRRL 5970, *Histoplasma* sp. (12 strains), *Lophophytongallinae* (*syn. Microsporum vanbreuseghemii*), *Microsporumferrugineum* (*syn. Trichophytonferrugineum*), *Nannizziopsisbarbatae*, *Arthrodermacrocatum* (*syn. Onygenacorvina*), *Ophidiomycesophidiicola*, *Trichophytonschoenleinii*, *Trichophytonsoudanense* and *Trichophytonviolaceum* (syn. *T.yaoundei*). All mitogenomes showed a conserved gene composition with 15 PCGs, two rRNAs and 22 to 26 tRNAs, except for *G.citrina* and *G.aurantiaca*, which showed 28 and 29 tRNAs, respectively.

The frequency of introns and open reading frames (ORF) related to putative HE elements within the newly-assembled mitogenomes also varied amongst species, ranging from 1–25 and 1–23, respectively (Suppl. material 3). The predicted intronic elements harbour completed or truncated ORFs, which are highly similar to homing endonucleases (HEG) of the LD and GIY families. The mitogenome nucleotide characterisation revealed a high bias towards the presence of A and T nucleotides, with values ranging from 72.96% in *Gymnascellacitrina* strain NRRL 5970 to 80.84% in *Paracoccidioidesamericana* strain Pb03 (Suppl. material 6).

### ﻿Genomic comparison amongst the newly- and previously-assembled *Onygenales* mitogenomes

The length of all 41 *Onygenales* mitochondrial genomes analysed ranged from 112,887 bp (*Paracoccidioidesamericana* strain Pb03) to 23,943 bp (*Microsporumcanis* strain ATCC 36299), both previously published (Wu et al. 2009; Misas et al. 2020). Amongst the five families analysed, the family *Gymnoascaceae* has the largest average length (60,950 ± 12,568.42 bp), considering only one representative genome per species (Suppl. material 7). The family *Ajellomycetaceae* has the most variable genome length (56,088.13 ± 25,880.74 bp), ranging from 31,579 bp (*Emmonsiacrescens* strain UAMH4076) to 112,887 bp (*Paracoccidioidesamericana*). A similar pattern was observed for intron total length, with the family *Onygenaceae* exhibiting the largest average (43,213.33 ± 10,432.96 bp) and *Ajellomycetaceae* the most variable size (27,286.12 ± 24,630.23 bp).

The mitogenomes coding portion (PCG + tRNA + rRNA) showed no variation in number of genes and length for the PCG and rRNA and a variation of the number of tRNAs ranging from 25 to 28 (Suppl. material 5). The intron size and the non-coding portion showed high variation in their length, providing evidence for their association with genome size. The mitogenomes were also characterised by a high AT content in all parts of the genome, with values greater than 70% (Suppl. material 6). Amongst the genomic regions, only tRNA and rRNA showed AT content values lower than 70%, although these values did not go below 60%.

### ﻿Mitochondrial gene re-arrangements

**Figure 2.**
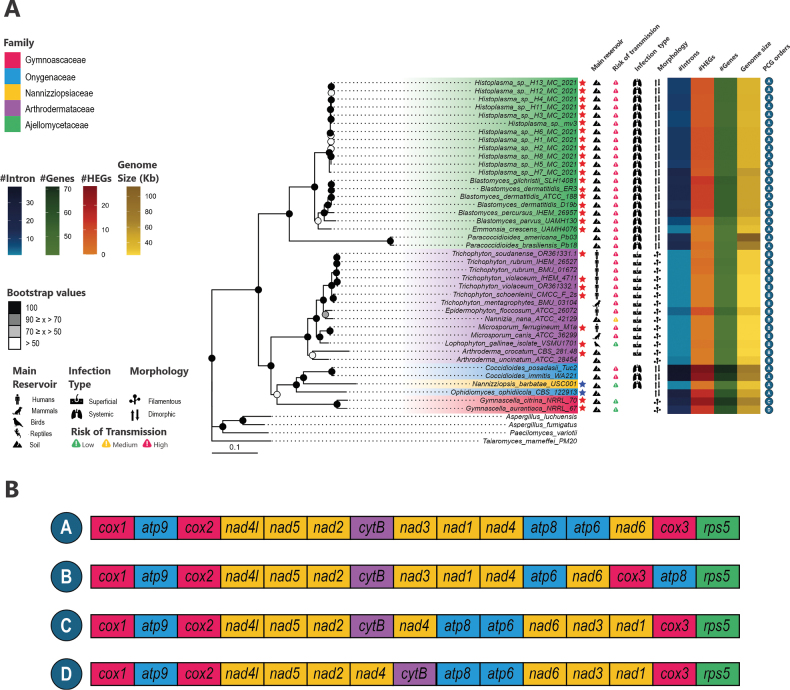
*Onygenales* phylogenetic proposition and its mitogenome conservation. **A.** Maximum Likelihood phylogenetic proposition using the codon alignment of the 15 mitochondrial PCGs. The number on the nodes represents the bootstrap values for 1,000 replicates. Each species possible hosts (general mammals - dog icon, humans - human icon, birds - bird icon, reptiles - lizard icon and soil - mountain icon), infection (cutaneous - skin icon or pulmonary/disseminated - lungs icon), morphology type are described using icons and risk of transmission from natural reservoirs are described using icons. The red stars mark the novel mitogenomes assembled herein and the blue stars mark the genomes assembled by Ladner et al. (2022) and Powell et al. (2023), which were also re-assembled and re-annotated in the present study; **B.** Synteny analysis of the 15 mitochondrial protein-coding genes (PCGs) identifies four distinct genomic configurations amongst the studied species. Colour-coded annotations demarcate the category of the genes as follows: red boxes correspond to genes encoding components of Complex IV (cytochrome c oxidase), blue to Complex V (*ATP* synthase), yellow to Complex I (*NADH* dehydrogenase), purple to Complex III (ubiquinol-cytochrome c reductase) and green to ribosomal protein genes.

We evaluate re-arrangements in the PCG order, as all these genes are located on the same DNA strand. Amongst the four types of gene re-arrangements (transposition, inverse transposition, inversion tandem duplication random loss), only transpositions were observed in *Onygenales* (Fig. 2B). This resulted in identifying four distinct PCG orders (Fig. 2B), with the A conformation shared by families *Ajellomycetaceae*, *Onygenaceae* and *Nannizziopsiaceae*. The *Arthrodermataceae* family underwent a transposition involving *ATP8* and *ATP6-NAD6-COX3*, resulting in conformation B. The family *Gymnoascaceae* exhibited two different conformations, labelled as conformation C and D. Our results yielded consistent confidence values for all nodes, except the *Onygenales* rooted node and the Gymnascella genera node.

### ﻿*Onygenales* phylogenetic proposition

The Maximum Likelihood phylogenetic tree proposed using the mitochondrial PCGs dataset recovered the monophyly of the *Ajellomycetaceae* and *Gymnoascaceae* families (Fig. 2A), with both branches receiving 100% bootstrap support. Notably, within the *Ajellomycetaceae* family, the *Paracoccidioides* branch yielded a branch length of 0.21.

The *Arthrodermataceae* family was included in a clade with *Onygenacorvina* strain CBS 281.48 (*Onygenaceae*). However, we hypothesise that the SRA data (NCBI Accession: SRR1705618) used to assemble this genome may be misidentified, as other studies have already described NCBI data annotated as *Onygenacorvina* that are, in fact, from *Arthrodermacrocatum* (Kandemir et al. 2022). Fungal species misidentification in public databases seems to be a common event, as can be exemplified by the NCBI Reference Sequence: NC_012830.1, which has the description as: *Arthrodermaobtusum ATCC 42129*, which is not accepted as the current name is described as *Nannizzianana*. Our analysis further confirms the representation as *N.nana*, as this species is grouped as the sister group of *Epidermophytonfloccosum* ATCC 26072 (as seen in Kandemir et al. (2022) and is distant from the other *Arthroderma* species.

A non-monophyletic clade containing three species from the *Onygenaceae* family (*Coccidioidesimmitis*, *Coccidioidesposadasii* and *Ophidiomycesophiodiicola*) and one species from the *Nannizziopsiaceae* family (*Nannizziopsisbarbatae*) was also recovered. Of particular interest is the observation that this clade grouped two species from different families that have reptiles as hosts and that have the same morphology (Yeast), *O.ophiodiicola* and *N.barbatae*, as sister species. The family *Nannizziopsiaceae* has previously been described as forming a cluster within *Onygenaceae*, thus representing a possible group of *Onygenaceae* rather than a separate family (Kandemir et al. 2022).

The analysis of the biological information within the phylogenetic tree reveals a relationship between the infectiousness and the species’ evolutionary development, as the inner groups showed a wider range of hosts, including humans as susceptible hosts. Similarly, the morphological type of the species is also in line with the evolutionary landscape. For instance, the dimorphic morphology has been described exclusively in the family *Ajellomycetaceae* and the genus *Coccidioides*, which is part of the family *Onygenaceae*.

The heat maps describing the genetic composition of the genomes indicate a phylogenetic signal in these characteristics, as closely-related species tend to resemble each other. The phylogenetic clusters have species with similar genetic patterns, such as the number of introns, HEGs and genes. However, the species of the genus *Paracoccidioides* constitute an exception to this pattern, as their large number of introns is not accomplished by a large number of HEGs in the genome. However, it is hypothesised that this discrepancy may be attributed to a misannotation issue. This assertion is supported by the observation that six ORFs were identified in the *P.brasiliensis* strain Pb18 and four ORFs were identified in the *Paracoccidioidesamericana* strain Pb03, both of which were not previously annotated in the NCBI genome database. These ORFs exhibited a relative similarity to elements previously identified in other fungal organisms (Suppl. material 8).

### ﻿*Onygenales* mitochondrial genome evolution patterns

**Figure 3.**
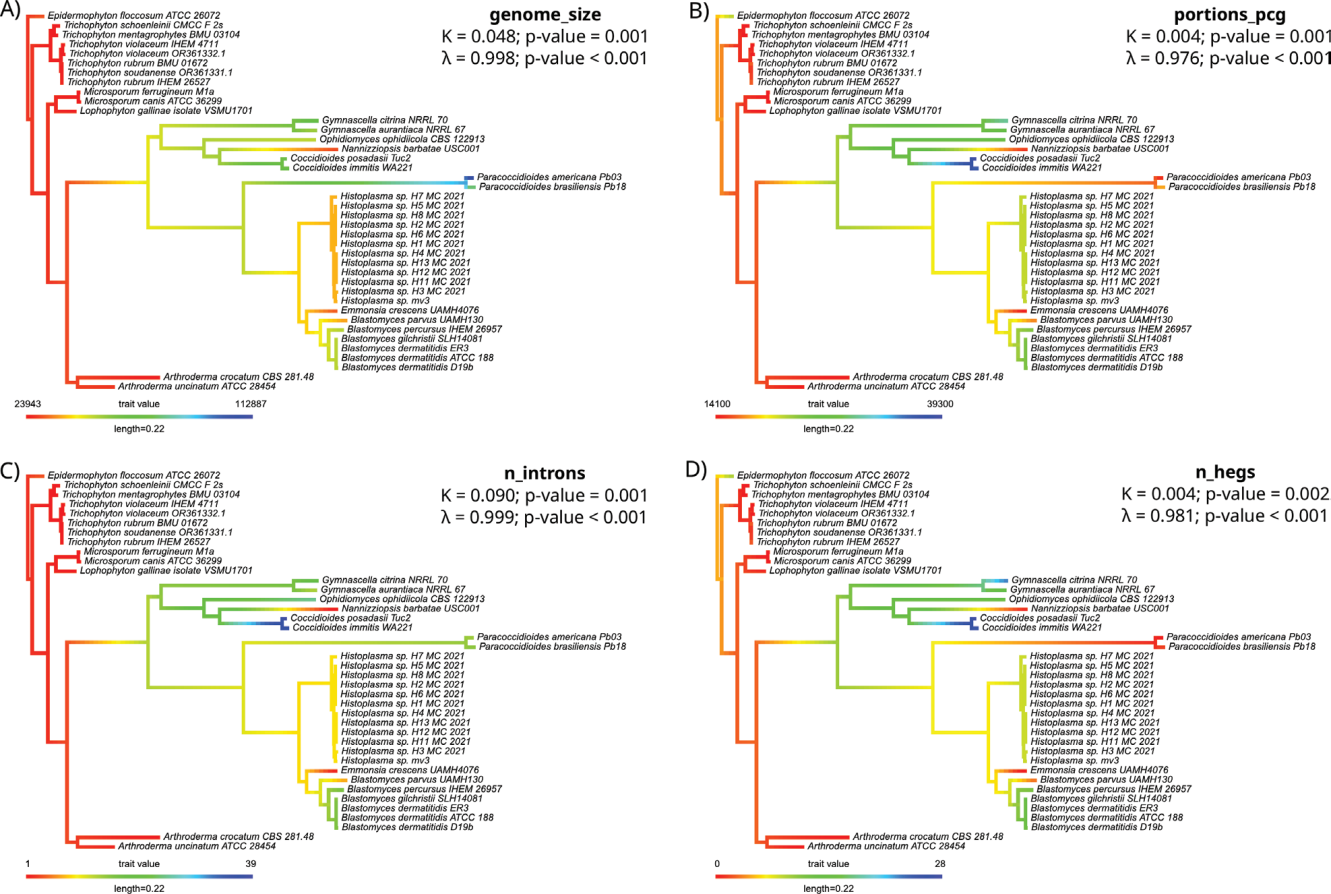
Phylogenetic distribution of mitogenomic traits across *Onygenales* species. **A.** Mitochondrial genome size; **B.** Proportion of protein-coding bases; **C.** Number of introns; **D.** Number of homing endonuclease genes (HEGs). Trait values are mapped on to the species tree using a heat-map gradient, revealing lineage-specific trends and phylogenetic structuring in mitochondrial genome architecture.

We analysed 13 structural traits from the mitochondrial genomes of 41 *Onygenales* species, including total genome size, gene counts (PCGs, tRNAs, rRNAs), intron and HEG numbers and genomic proportions (coding, non-coding and intronic regions). Phylogenetic signal analyses revealed that genome size, total gene number, intron number, HEG number and intronic proportion exhibited significant phylogenetic structure. Values of Blomberg’s K and Pagel’s λ were significantly greater than zero and often near one, indicating a moderate to strong phylogenetic signal with some evolutionary lability (Fig. 3).

**Figure 4.**
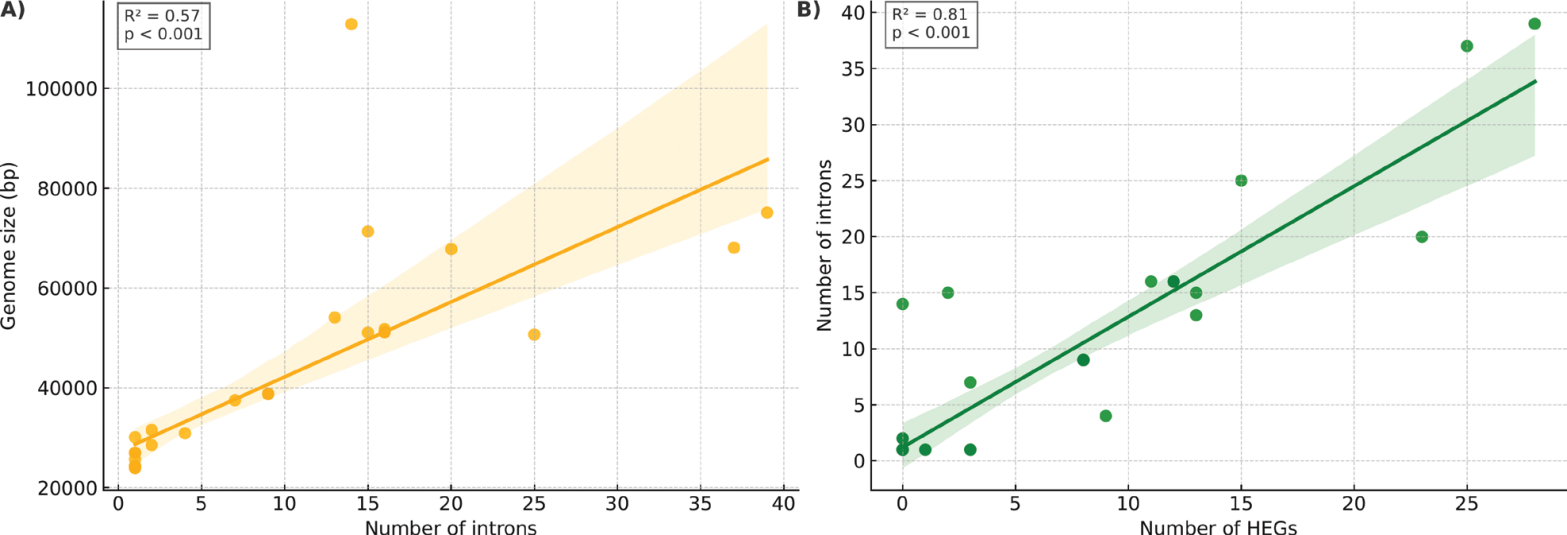
Correlation between intron number and HEG abundance in *Onygenales* mitogenomes. **A.** Scatterplot showing the relationship between the number of introns and number of HEGs, with regression line and confidence interval estimated via Phylogenetic Generalised Least Squares (PGLS); **B.** Statistical summary of the regression model, including coefficient of determination (R²) and p-value, indicating significant positive correlation and supporting the hypothesis of intron–HEG co-evolution as a driver of mitogenome expansion.

Phylogenetic Generalised Least Squares (PGLS) regressions revealed a strong and significant positive correlation between intron count and HEG number (R² = 0.64, p < 0.001), providing statistical support for a hypothesis of coordinated evolution between these two genomic features (Fig. 4). In addition, both the proportion of intronic regions and the number of HEGs exhibited a significant correlation with total mitochondrial genome size, suggesting that these components are major contributors to genome expansion in *Onygenales*.

The Mantel test, comparing phylogenetic distances with Euclidean distances, based on trait data, yielded a significant result (p = 0.004), indicating that closely-related species tend to share similar mitogenomic structural profiles. This supports the hypothesis that mitochondrial genomic architecture is non-randomly distributed and phylogenetically structured. Notably, exceptions to these patterns were found in the genus *Paracoccidioides*, which harbours large mitochondrial genomes with high intron content, but relatively few annotated HEGs.

### ﻿Nucleotide diversity and gene selection

The mean nucleotide diversity value of all 15 PCGs for the 41 *Onygenales* mitogenomes analysed in this work was 0.140, ranging from 0.059 to 0.325 (Suppl. material 10). Two PCGs showed nucleotide diversity values higher than twice the median value of the overall diversity: *NAD6* and *RPS5* (Suppl. material 10). Furthermore, the omega (ratio of non-synonymous per synonymous substitutions) values of all PCGs ranged from 0.022 (*NAD4l*) to 0.0904 (*RPS5*), indicating an overall pattern of negative selection (Suppl. material 10).

## ﻿Discussion

This study contributes to the expansion of the mitochondrial genomic landscape of *Onygenales* by providing 30 newly-assembled mitogenomes and applying comparative phylogenetic approaches to test two central hypotheses: (1) that structural traits of fungal mitogenomes are phylogenetically conserved within the order and (2) that introns and homing endonuclease genes (HEGs) have co-evolved, driving mitochondrial genome expansion in *Onygenales*.

Our analyses revealed strong phylogenetic signals for several mitogenomic traits, including total genome size, gene number, intron count, HEG abundance and the proportion of intronic regions. These findings support the hypothesis that certain genomic features are phylogenetically conserved across *Onygenales*. Blomberg’s K and Pagel’s λ values consistently indicated that closely-related species share similar mitogenomic architectures. This finding suggests that evolutionary history plays a central role in shaping mitochondrial genome composition. Importantly, the Mantel test further confirmed that species with lower phylogenetic distances exhibit greater overall genomic similarity (p = 0.004), thereby reinforcing the presence of phylogenetically structured variation. A similar set of results has been reported in other fungal groups, such as *Eurotiales* and *Hypocreales* (Aguileta et al. 2014; Korovesi et al. 2018). In these cases, the conservation of gene content and organisation was observed within families.

The stability of protein-coding gene content across the 41 *Onygenales* mitogenomes analysed here, including the presence of 15 core PCGs and two rRNAs, reflects a conserved mitochondrial core function essential for oxidative phosphorylation and metabolic regulation (Herst et al. 2017; Fonseca et al. 2020). However, variation in non-coding and intronic regions was substantial and this variation was not randomly distributed, but instead reflected phylogenetic structure. For instance, the *Gymnoascaceae* family showed the highest intron and HEG counts, while the *Ajellomycetaceae* family exhibited the widest range of genome sizes, suggesting lineage-specific expansions. These results align with earlier work by Burger et al. (2003) and Liu et al. (2020), which emphasised the dynamic nature of fungal mitogenomes, particularly in non-coding regions. Collectively, these results robustly confirm our first hypothesis. We show for the first time in *Onygenales* that multiple structural features of the mitogenome exhibit statistically significant phylogenetic signals, indicating vertical inheritance and evolutionary constraint.

Our study provides empirical evidence supporting the coordinated evolution of group I introns and their associated homing endonuclease genes (HEGs) in *Onygenales*, as previously hypothesised in the “aenaon” model proposed by Megarioti and Kouvelis (2020). According to this model, the relationship between introns and HEGs is not merely functional or incidental, but reflects a perpetual and dynamic co-evolution involving recombination, gene fusion and horizontal gene transfer (HGT). These composite elements, self-splicing introns harbouring endonuclease-encoding ORFs, form mobile units that have shaped fungal mitochondrial genome architecture over evolutionary timescales.

In *Onygenales*, a robust and statistically-significant positive correlation was observed between the number of introns and the number of HEGs (R² = 0.64; p < 0.001), consistent with the premise that HEGs contribute to the retention and spread of introns (Goddard and Burt 1999; Belfort et al. 2002). This pattern persisted even after controlling for phylogenetic relatedness using PGLS, reinforcing the hypothesis of co-evolution rather than coincidental co-occurrence. Moreover, traits, such as genome size and intronic proportion, were tightly linked to both intron and HEG abundance, suggesting that their proliferation has been a major force in mitochondrial genome expansion within this particular fungal order.

Our data resonate with several aspects of the “aenaon” model. Initially, it was determined that HEGs are consistently embedded within intronic loops that do not interfere with catalytic splicing functions, an arrangement that allows cis-acting mobility with minimal host cost (Megarioti and Kouvelis 2020). Secondly, the clear phylogenetic structuring of these traits across *Onygenales* species, alongside outlier cases (e.g. *Paracoccidioides* spp.), suggests that episodic intron-HEG invasions have occurred throughout the evolution of *Onygenales*. This finding is consistent with both “intron-early” and “intron-late” processes described in the mitochondrial history of fungi (Lang et al. 2007; Zubaer et al. 2018).

Notably, the intron types most commonly harbouring HEGs in *Onygenales*, such as group IB and ID, are also identified in Megarioti and Kouvelis (2020) as ancestral insertion targets, further supporting the hypothesis that these subtypes represent conserved hotspots for composite element integration. The variability we observed in intron and HEG presence across families, especially within *Gymnoascaceae* and *Ajellomycetaceae*, suggests that lineage-specific events, including HEG degeneration, intron loss or recombination-mediated re-arrangements, continue to shape mitochondrial genome diversity even within this order. Altogether, these findings provide a compelling validation of our second hypothesis: that introns and HEGs have evolved in a coordinated fashion and act as major contributors to mitogenome expansion in *Onygenales*. The statistical and comparative evidence presented here places *Onygenales* within the broader evolutionary framework described by the “aenaon” model.

The mitochondrial phylogeny inferred from protein-coding genes provided a well-supported framework that largely agrees with previous nuclear-based phylogenies for *Onygenales* (Kandemir et al. 2022). Previous studies have also employed mitogenomes for resolving phylogenetic relationships within *Onygenales*. However, these investigations were either focused on intraspecific relationships within the *Coccidioides* genus (Teixeira et al. 2021) or utilised a limited taxonomic sampling of *Onygenales* representatives (Zhang et al. 2024). In both studies, mtDNA proved effective in delimiting intra-genus relationships, though it failed to provide consistent resolution amongst intraspecific strains of *Coccidioides*, possibly due the admixture events from hybridisation or interspecies introgression, which was also observed in *Paracoccidioides*. Notably, mtDNA successfully resolved relationships within the *Arthrodermataceae* family in Zhang et al. (2024). These findings suggest that mitochondrial genomes hold promise for resolving intra-ordinal phylogenetic relationships, a conclusion further supported by our results using an expanded dataset. Our tree recovered the monophyly of *Ajellomycetaceae* and *Gymnoascaceae* with strong bootstrap support, while indicating non-monophyly for *Onygenaceae* and *Arthrodermataceae*. Notably, *Onygenacorvina* (strain CBS 281.48), placed within *Arthrodermataceae* in our reconstruction, is likely misidentified, making the *Arthrodermataceae* also monophyletic. Prior studies have revealed inconsistencies in its NCBI assignment, with several sequences labelled as *O.corvina* later re-assigned to *Arthrodermacrocatum* (Kandemir et al. 2022). Similarly, *Arthrodermaobtusum* (NC_012830.1) appears better supported as *Nannizzianana*, forming a clade with *Epidermophytonfloccosum*.

These findings reinforce the efficacy of mitogenomic data in identifying taxonomic inconsistencies and refining species-level assignments. The placement of *Nannizziopsisbarbatae* within *Onygenaceae* also corroborates previous proposals to subsume *Nannizziopsiaceae* into *Onygenaceae* (Stchigel et al. 2013; Kandemir et al. 2022). Interestingly, the close relationship between *N.barbatae* and *Ophidiomycesophiodiicola*, both reptile-associated yeast-like fungi, may reflect shared ecological adaptations, though such patterns must be interpreted cautiously given the limited sampling.

The tree topology also highlighted phylogenetic structuring of ecological traits. For instance, dimorphic morphology was restricted to *Ajellomycetaceae* and *Coccidioides*, supporting the independent evolution of this trait. The placing anthropophilic and zoophilic *Trichophyton* species in distinct clades echoes previous nuclear reconstructions (Cornet et al. 2021; Kabtani and Ranque 2023), although the *Trichophyton* clade has already been presented as a polyphyletic group composed of four species series recognised (A1-4) by molecular studies and expanded sampling of isolated and species, with no determinants of anthropophilic and zoophilic species, of which it should be carefully addressed with updated clinical data for more precise description (de Hoog et al. 2017). Here, a similar structure was also found, with the *T.mentagrophytes* BMU 03104 clustered with *T.schoenleinii* CMCC F2S. Furthermore, the *Trichophytonsoudanense* mitogenome assembled here provides the first mitochondrial resource for this species and shows clear differentiation from *T.violaceum* and *T.rubrum*, further validating its species-level status. This highlights the potential of mitochondrial phylogenomic data to resolve complex taxonomic questions, especially when paired with ecological and morphological traits.

By increasing the number of available *Onygenales* mitogenomes by over 270%, this study provides a foundation for future evolutionary, ecological and molecular investigations in the group. The identification of *NAD6* and *RPS5* as intron-free, highly variable genes also opens avenues for their use as complementary mitochondrial barcodes, especially given the limitations of *COX1* due to extensive intron content (Santamaria et al. 2009; Schoch et al. 2012). The consistent phylogenetic structure exhibited by these traits highlights their utility in evolutionary inferences and species delimitation.

Future studies could further refine the mechanistic understanding of intron and HEG propagation, explore the role of recombination in gene order re-arrangements and assess the ecological correlates of mitogenomic expansion. Furthermore, expanding the taxon sampling, particularly amongst under-represented *Onygenales* families, will enhance the resolution of phylogenetic and functional inferences.

## ﻿Conclusions

This study contributes to the expansion of available mitogenomic resources for *Onygenales* and provides new insights into the evolutionary dynamics of fungal mitochondrial genomes. Our analyses demonstrate that key structural traits, such as gene content, genome size, intron abundance and HEG presence, exhibit significant phylogenetic signals, supporting their evolutionary conservation across lineages. Furthermore, evidence is presented demonstrating that introns and homing endonuclease genes have co-evolved in a correlated manner, contributing to genome size variation, a pattern consistent with the “aenaon” model of intron-HEG co-evolution. In addition, our phylogenetic reconstruction, based on mitochondrial genes, clarified taxonomic inconsistencies and corroborated established ecological and morphological patterns. Collectively, these findings establish a genomic and evolutionary framework for further studies on mitochondrial function, mobility and diversity within *Onygenales*.

## ﻿Additional information

### Adherence to national and international regulations

All the fungal strains used in this study have been legally obtained, respecting the Convention on Biological Diversity (Rio Convention).

### Author ORCIDs

Héctor Antônio Assunção Romão https://orcid.org/0000-0002-3137-1336

Thalison Rodrigues Moreira https://orcid.org/0009-0008-3265-0493

Leonardo Carlos Jeronimo Corvalán https://orcid.org/0000-0002-3945-4208

Amanda Alves de Melo-Ximenes https://orcid.org/0000-0001-6873-7322

Alexandre Melo Bailão https://orcid.org/0000-0001-7754-4047

Clayton Luiz Borges https://orcid.org/0000-0002-9914-8771

Renata de Oliveira Dias https://orcid.org/0000-0001-6729-5980

Rhewter Nunes https://orcid.org/0000-0001-7770-7707

### Data availability

All of the data that support the findings of this study are available in the main text or Supplementary Information.

## Supplementary materials

Supplementary material 1 Description of the *Onygenales* mitochondrial resources already available before the current study Data type xlsx https://binary.pensoft.net/file/1376178This dataset is made available under the Open Database License (http://opendatacommons.org/licenses/odbl/1.0/). The Open Database License (ODbL) is a license agreement intended to allow users to freely share, modify, and use this Dataset while maintaining this same freedom for others, provided that the original source and author(s) are credited.Héctor Antônio Assunção Romão, Thalison Rodrigues Moreira, Leonardo Carlos Jeronimo Corvalán, Amanda Alves de Melo-Ximenes, Alexandre Melo Bailão, Clayton Luiz Borges, Renata de Oliveira Dias, Rhewter Nunes

Supplementary material 2 Description of the data used to assemble the *Onygenales* mitogenomes Data type xlsx https://binary.pensoft.net/file/1376179This dataset is made available under the Open Database License (http://opendatacommons.org/licenses/odbl/1.0/). The Open Database License (ODbL) is a license agreement intended to allow users to freely share, modify, and use this Dataset while maintaining this same freedom for others, provided that the original source and author(s) are credited.Héctor Antônio Assunção Romão, Thalison Rodrigues Moreira, Leonardo Carlos Jeronimo Corvalán, Amanda Alves de Melo-Ximenes, Alexandre Melo Bailão, Clayton Luiz Borges, Renata de Oliveira Dias, Rhewter Nunes

Supplementary material 3 AWA metrics retrieved for each complete genome assembled Data type xlsx https://binary.pensoft.net/file/1376180This dataset is made available under the Open Database License (http://opendatacommons.org/licenses/odbl/1.0/). The Open Database License (ODbL) is a license agreement intended to allow users to freely share, modify, and use this Dataset while maintaining this same freedom for others, provided that the original source and author(s) are credited.Héctor Antônio Assunção Romão, Thalison Rodrigues Moreira, Leonardo Carlos Jeronimo Corvalán, Amanda Alves de Melo-Ximenes, Alexandre Melo Bailão, Clayton Luiz Borges, Renata de Oliveira Dias, Rhewter Nunes

Supplementary material 4 Articles used to identify morphological and infection-related characteristics of the species Data type xlsx https://binary.pensoft.net/file/1376181This dataset is made available under the Open Database License (http://opendatacommons.org/licenses/odbl/1.0/). The Open Database License (ODbL) is a license agreement intended to allow users to freely share, modify, and use this Dataset while maintaining this same freedom for others, provided that the original source and author(s) are credited.Héctor Antônio Assunção Romão, Thalison Rodrigues Moreira, Leonardo Carlos Jeronimo Corvalán, Amanda Alves de Melo-Ximenes, Alexandre Melo Bailão, Clayton Luiz Borges, Renata de Oliveira Dias, Rhewter Nunes

Supplementary material 5 Genome characteristics of each *Onygenales* mitochondrial genome analysed in this study Data type xlsx https://binary.pensoft.net/file/1376182This dataset is made available under the Open Database License (http://opendatacommons.org/licenses/odbl/1.0/). The Open Database License (ODbL) is a license agreement intended to allow users to freely share, modify, and use this Dataset while maintaining this same freedom for others, provided that the original source and author(s) are credited.Héctor Antônio Assunção Romão, Thalison Rodrigues Moreira, Leonardo Carlos Jeronimo Corvalán, Amanda Alves de Melo-Ximenes, Alexandre Melo Bailão, Clayton Luiz Borges, Renata de Oliveira Dias, Rhewter Nunes

Supplementary material 6 Base composition metrics of each genetic element within the mtDNA of *Onygenales* species Data type xlsx https://binary.pensoft.net/file/1376183This dataset is made available under the Open Database License (http://opendatacommons.org/licenses/odbl/1.0/). The Open Database License (ODbL) is a license agreement intended to allow users to freely share, modify, and use this Dataset while maintaining this same freedom for others, provided that the original source and author(s) are credited.Héctor Antônio Assunção Romão, Thalison Rodrigues Moreira, Leonardo Carlos Jeronimo Corvalán, Amanda Alves de Melo-Ximenes, Alexandre Melo Bailão, Clayton Luiz Borges, Renata de Oliveira Dias, Rhewter Nunes

Supplementary material 7 Genome size and structure metrics for each *Onygenales* family represented in the dataset Data type xlsx https://binary.pensoft.net/file/1376184This dataset is made available under the Open Database License (http://opendatacommons.org/licenses/odbl/1.0/). The Open Database License (ODbL) is a license agreement intended to allow users to freely share, modify, and use this Dataset while maintaining this same freedom for others, provided that the original source and author(s) are credited.Héctor Antônio Assunção Romão, Thalison Rodrigues Moreira, Leonardo Carlos Jeronimo Corvalán, Amanda Alves de Melo-Ximenes, Alexandre Melo Bailão, Clayton Luiz Borges, Renata de Oliveira Dias, Rhewter Nunes

Supplementary material 8 Description of the new ORFs predicted for the two Paracoccidioides species Data type xlsx https://binary.pensoft.net/file/1376185This dataset is made available under the Open Database License (http://opendatacommons.org/licenses/odbl/1.0/). The Open Database License (ODbL) is a license agreement intended to allow users to freely share, modify, and use this Dataset while maintaining this same freedom for others, provided that the original source and author(s) are credited.Héctor Antônio Assunção Romão, Thalison Rodrigues Moreira, Leonardo Carlos Jeronimo Corvalán, Amanda Alves de Melo-Ximenes, Alexandre Melo Bailão, Clayton Luiz Borges, Renata de Oliveira Dias, Rhewter Nunes

Supplementary material 9 Results of phylogenetic signal (Blomberg’s K and Pagel’s λ), PGLS regressions and Mantel tests applied to mitochondrial genomic traits Data type xlsx https://binary.pensoft.net/file/1376186This dataset is made available under the Open Database License (http://opendatacommons.org/licenses/odbl/1.0/). The Open Database License (ODbL) is a license agreement intended to allow users to freely share, modify, and use this Dataset while maintaining this same freedom for others, provided that the original source and author(s) are credited.Héctor Antônio Assunção Romão, Thalison Rodrigues Moreira, Leonardo Carlos Jeronimo Corvalán, Amanda Alves de Melo-Ximenes, Alexandre Melo Bailão, Clayton Luiz Borges, Renata de Oliveira Dias, Rhewter Nunes

Supplementary material 10 Evolutive genetic comparative analysis along 41 *Onygenales* mitogenomes Data type xlsx Explanation note **A.** Values of nucleotide diversity (π) along the 15 PCGs. The red line represents twice the median value of nucleotide diversity and the two highlighted PCG have nucleotide diversity values higher than twice the mean; **B.** Omega values for each PCG. https://binary.pensoft.net/file/1376187This dataset is made available under the Open Database License (http://opendatacommons.org/licenses/odbl/1.0/). The Open Database License (ODbL) is a license agreement intended to allow users to freely share, modify, and use this Dataset while maintaining this same freedom for others, provided that the original source and author(s) are credited.Héctor Antônio Assunção Romão, Thalison Rodrigues Moreira, Leonardo Carlos Jeronimo Corvalán, Amanda Alves de Melo-Ximenes, Alexandre Melo Bailão, Clayton Luiz Borges, Renata de Oliveira Dias, Rhewter Nunes
