# Supplementary figures and images for: ﻿Phylogenetic and evolutionary insights from 30 newly-assembled Onygenales Mitochondrial Genomes: co-evolution of introns and HEGs shapes mitogenome size variation
Source: IMA Fungus. 2025 Jul 17;16:e150451. doi: 10.3897/imafungus.16.150451 (PMC12290462; doi:10.3897/imafungus.16.150451)

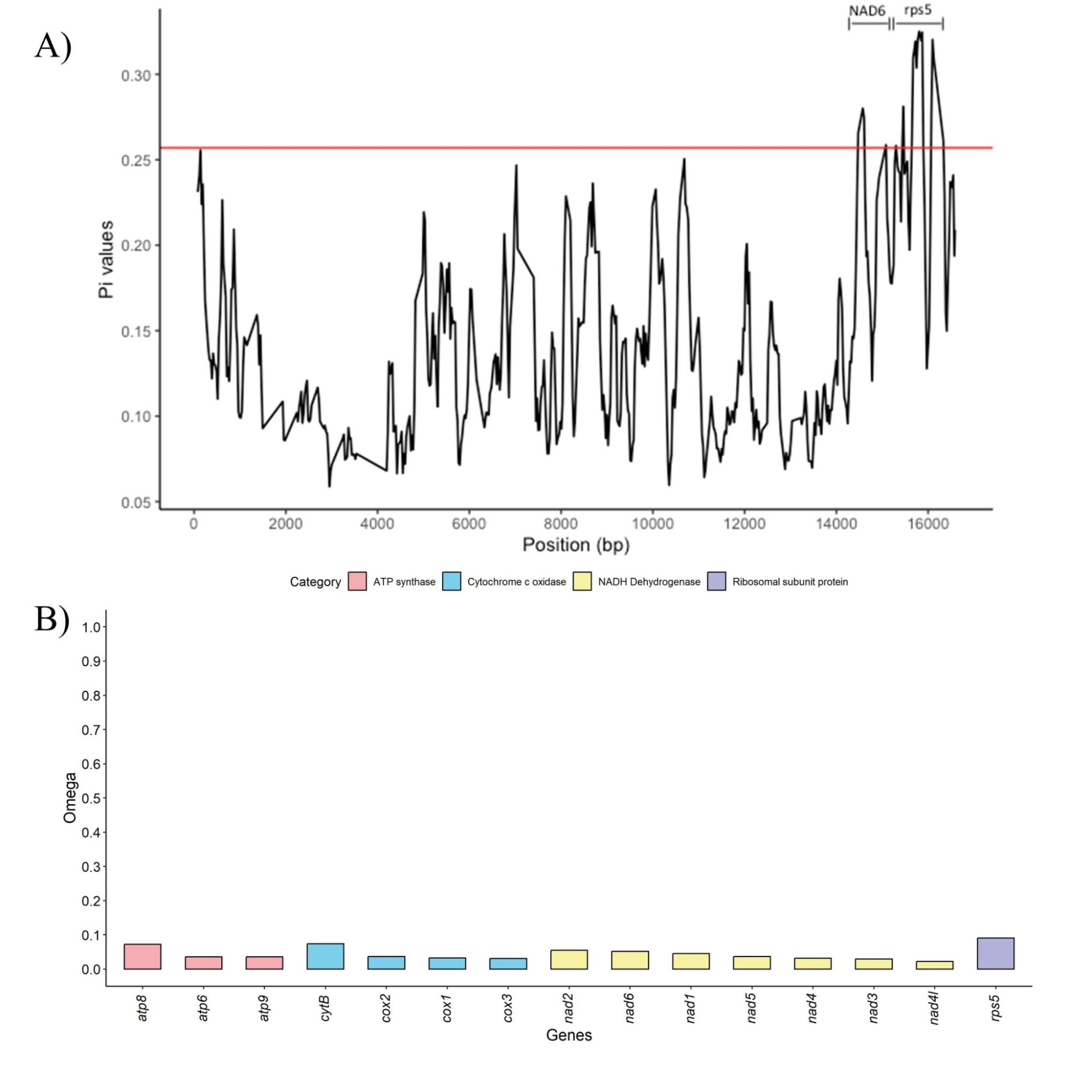

Supplement: Supplementary material 10 — Evolutive genetic comparative analysis along 41 Onygenales mitogenomes [file imafungus-16-e150451-s010.jpg]
